# Supplementary material for: Energy-saving hydrogen production by seawater electrolysis coupling tip-enhanced electric field promoted electrocatalytic sulfion oxidation
Source: Nat Commun. 2024 Jul 22;15:6173. doi: 10.1038/s41467-024-49931-5 (PMC11263359; doi:10.1038/s41467-024-49931-5)
Supplement: Supplementary file 1 — Supplementary Information [file 41467_2024_49931_MOESM1_ESM.pdf]

**Supplementary Information**

# Energy-Saving Hydrogen Production by Seawater Electrolysis Coupling Tip-Enhanced Electric Field Promoted Electrocatalytic Sulfion Oxidation

Tongtong Li[1], Boran Wang[1], Yu Cao[2], Zhexuan Liu[1], Shaogang Wang[3], Qi Zhang[1], Jie Sun[2], Guangmin Zhou[1]*

[1]Tsinghua-Berkeley Shenzhen Institute & Tsinghua Shenzhen International Graduate School, Tsinghua University, Shenzhen 518055, P. R. China.

[2]School of Chemical Engineering and Technology, Tianjin University, Tianjin 300072, P. R. China.

[3]Shenyang National Laboratory for Materials Science, Institute of Metal Research, Chinese Academy of Sciences, Shenyang 110016, P. R. China.

*Corresponding E-mail: guangminzhou@sz.tsinghua.edu.cn

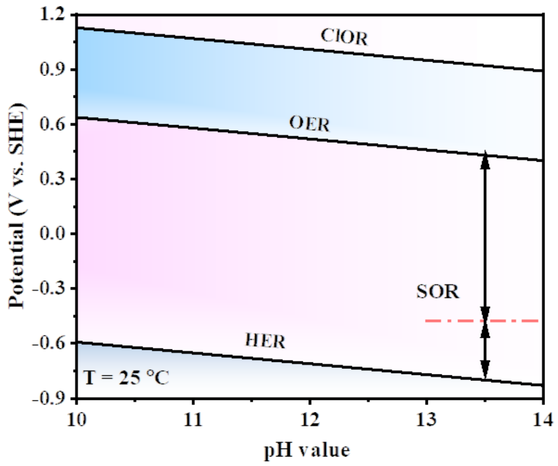


Supplementary Figure 1 | Pourbaix diagram of the SOR, OER, HER, and ClOR in pH 10-14.

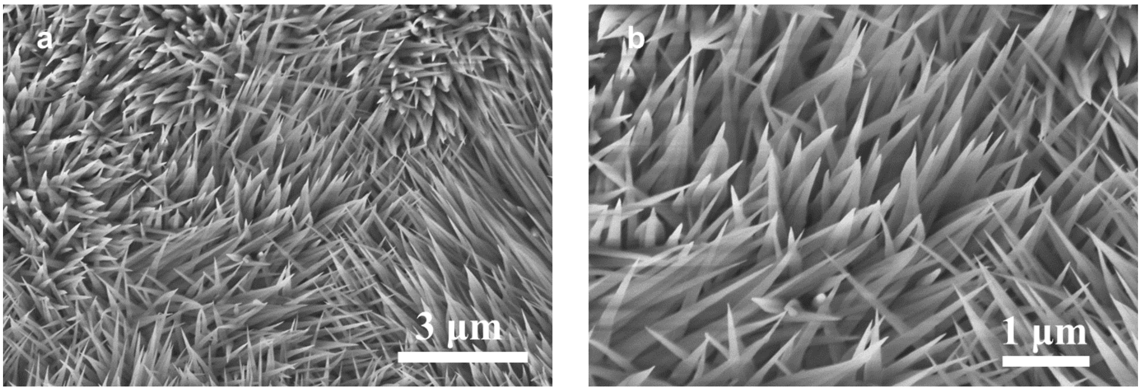


Supplementary Figure 2 | (a, b) SEM images of *n*-$Co(OH)_2$@NF.

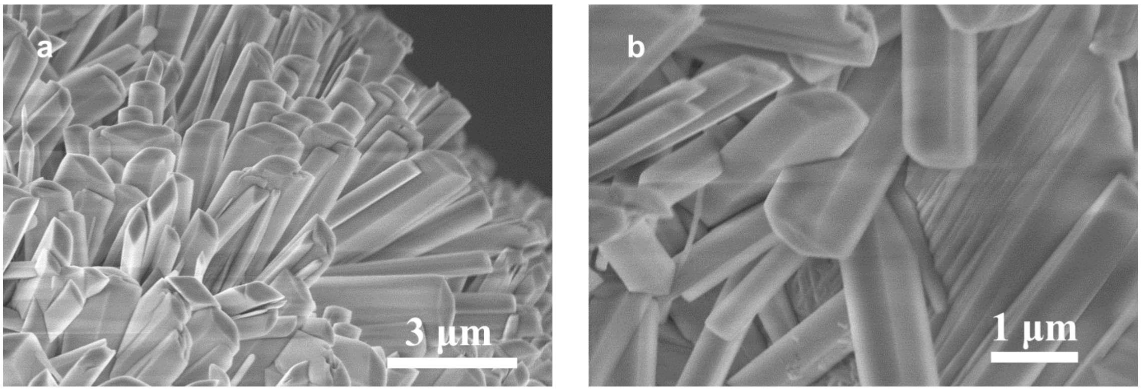


Supplementary Figure 3 | (a, b) SEM images of *r*-$Co(OH)_2$@NF.

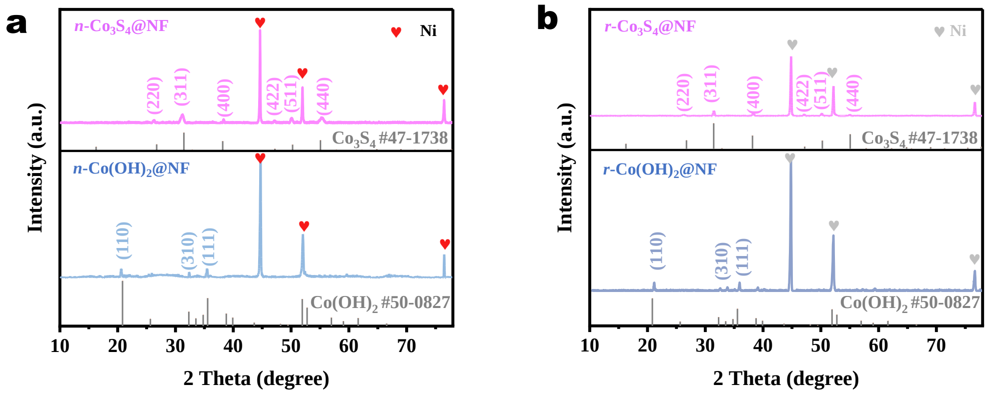


Supplementary Figure 4 | (a) XRD patterns of *n*-$Co(OH)_2$@NF and *n*-$Co_3S_4$@NF. (b) XRD patterns of *r*-$Co(OH)_2$@NF and *r*-$Co_3S_4$@NF.

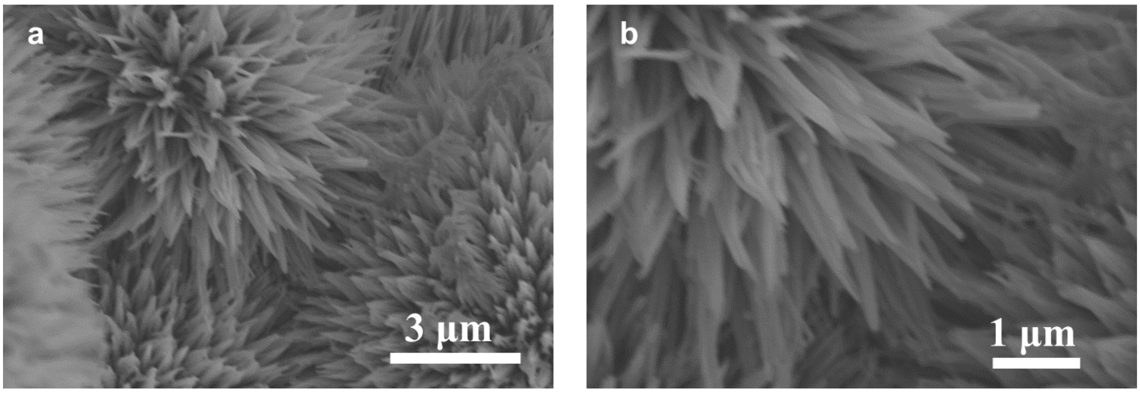


Supplementary Figure 5 | (a, b) SEM images of *n*-$Co_3S_4$@NF.

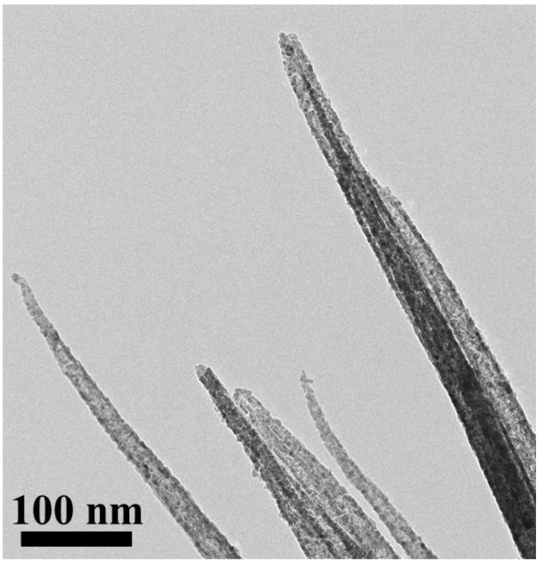


Supplementary Figure 6 | TEM image of $n$-$Co_3S_4$.

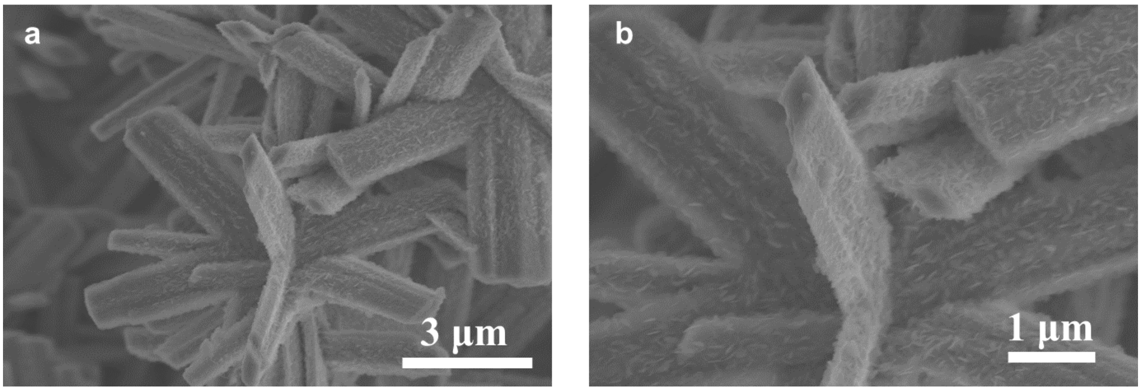


Supplementary Figure 7 | (a, b) SEM images of *r*-$Co_3S_4$@NF.

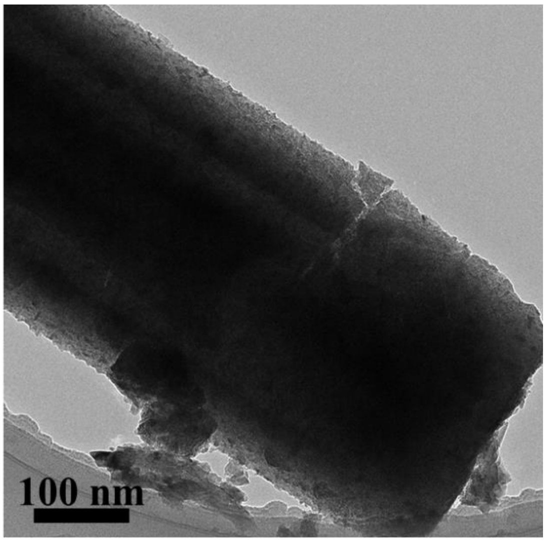


Supplementary Figure 8 | TEM image of *r*-$Co_3S_4$.

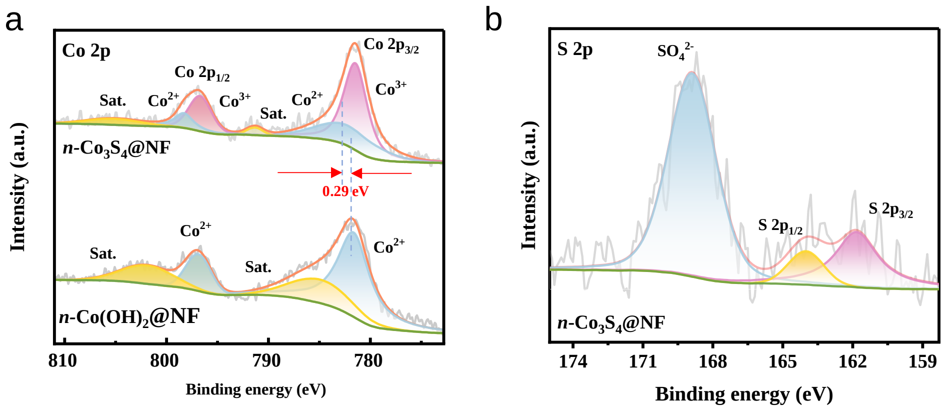


Supplementary Figure 9 | (a) High-resolution Co 2*p* XPS spectrum of *n*-$Co(OH)_2$@NF and *n*-$Co_3S_4$@NF. (b) High-resolution S 2*p* XPS spectrum of *n*-$Co_3S_4$@NF.

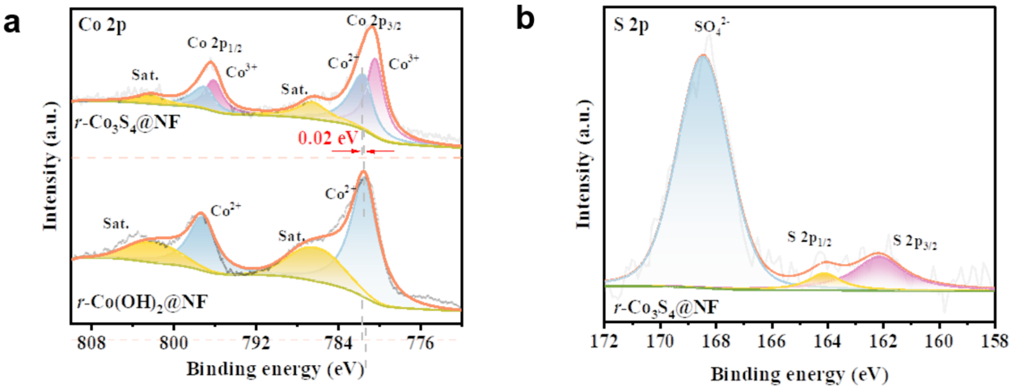


Supplementary Figure 10 | (a) High-resolution Co 2*p* XPS spectrum of *r*-$Co(OH)_2$@NF and *r*-$Co_3S_4$@NF. (b) High-resolution S 2*p* XPS spectrum of *r*-$Co_3S_4$@NF.

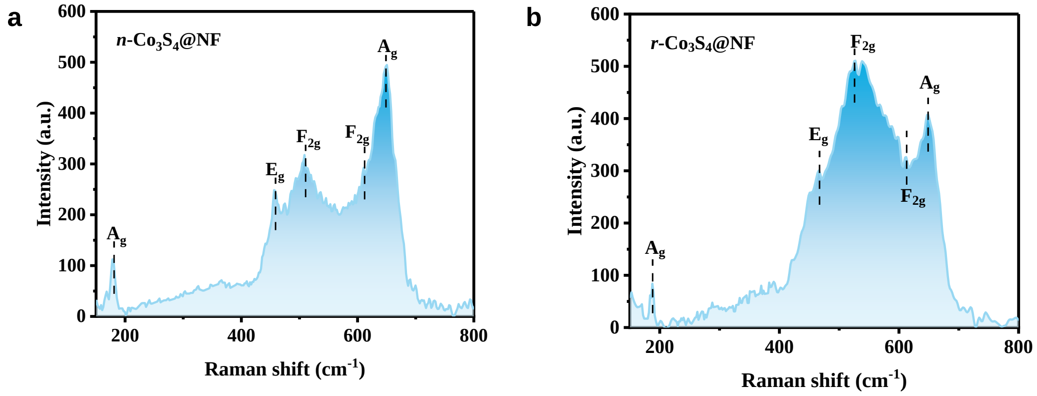


Supplementary Figure 11 | Raman spectra of (a) *n*-$Co_3S_4$@NF and (b) *r*-$Co_3S_4$@NF.

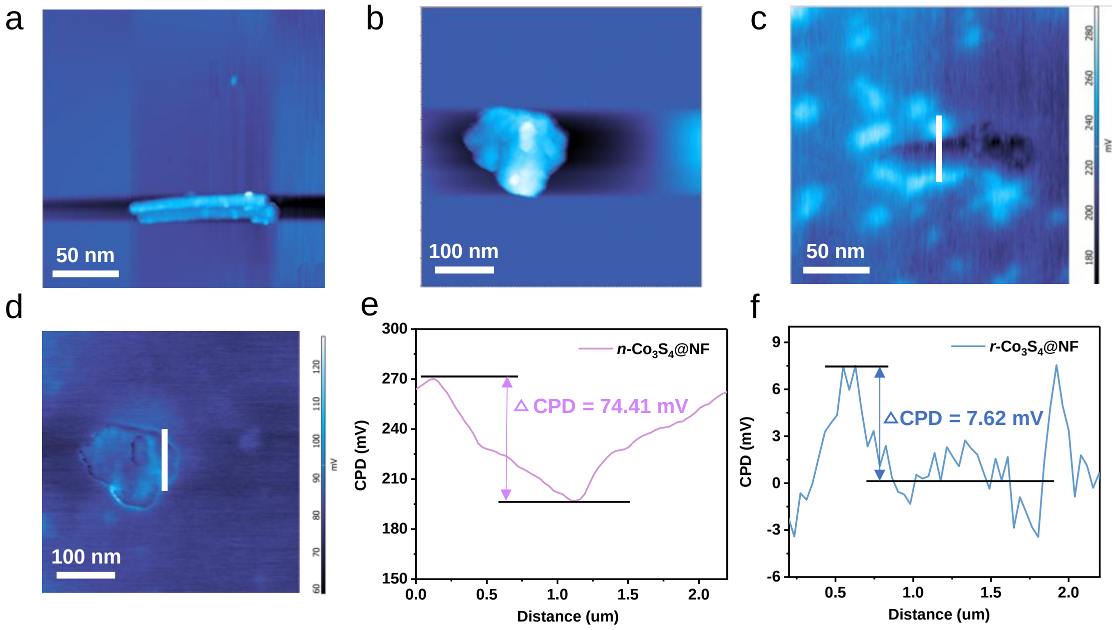


Supplementary Figure 12 | Topography images of (a) *n*-$Co_3S_4$ and (b) *r*-$Co_3S_4$; Surface potential images of (c) *n*-$Co_3S_4$ and (d) *r*-$Co_3S_4$. Contact potential difference of (e) *n*-$Co_3S_4$ and (f) *r*-$Co_3S_4$.

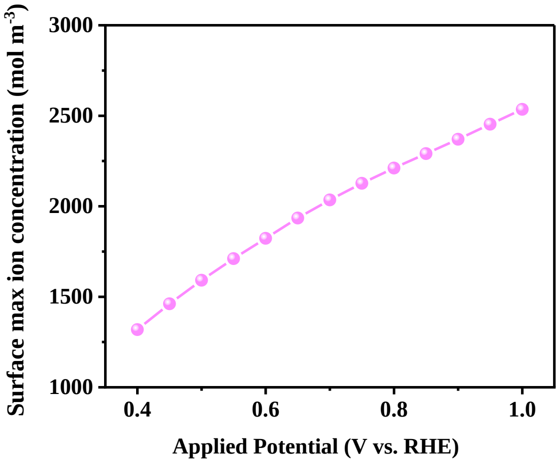


Supplementary Figure 13 | Relationship between applied voltage and surface maximum ion concentration in Comsol simulations.

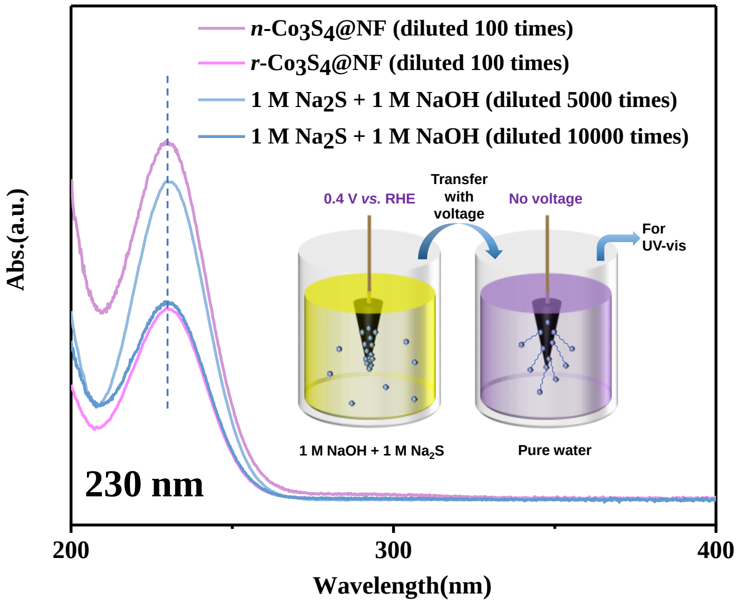


Supplementary Figure 14 | $S^{2-}$ absorbing test.

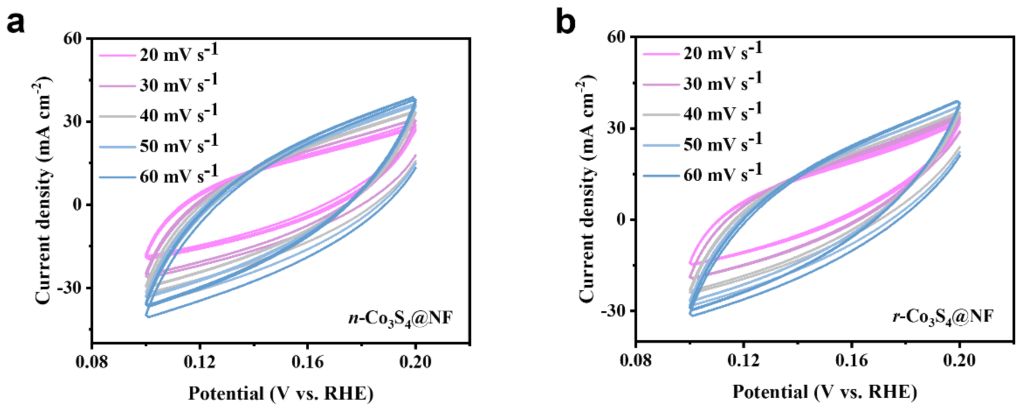


Supplementary Figure 15 | The CV curves under different scan rates in the non-Faraday region of (a)*n*-$Co_3S_4$@NF and (b)*r*-$Co_3S_4$@NF.

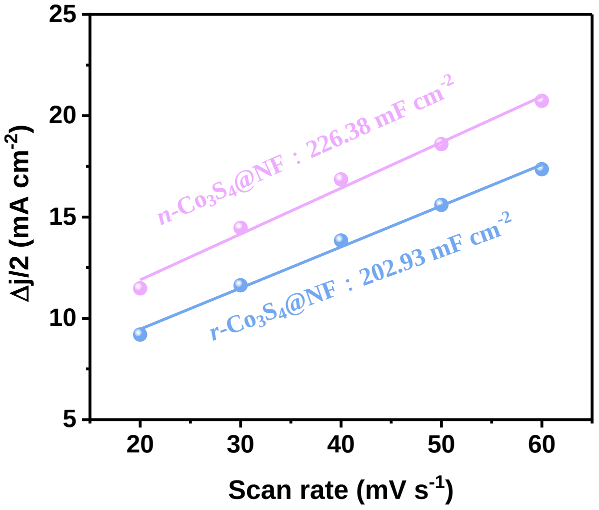


Supplementary Figure 16 | $C_{dl}$ of catalysts derived from the current density versus the scan rate.

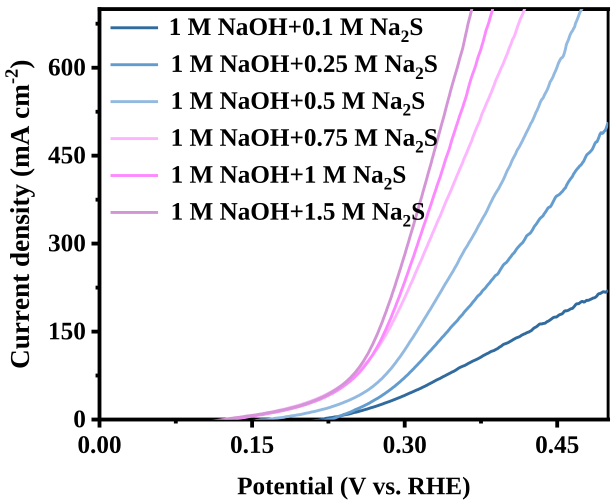


Supplementary Figure 17 | LSV curves of *n*-$Co_3S_4$@NF for SOR in different concentrations of $Na_2S$.

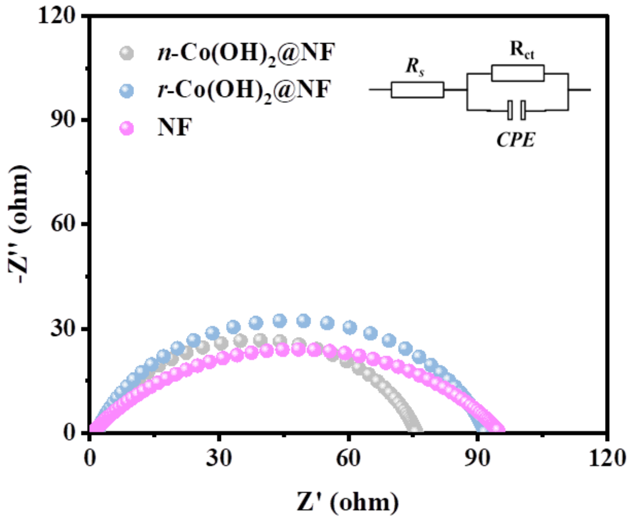


Supplementary Figure 18 | EIS plots of *n*-$Co(OH)_2$@NF, *r*-$Co(OH)_2$@NF, and NF for SOR in 1 M $Na_2S$ + 1 M NaOH electrolyte.

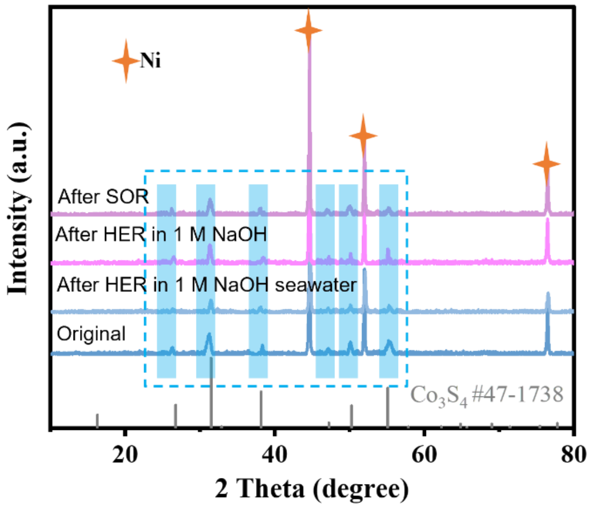


Supplementary Figure 19 | XRD patterns of *n*-$Co_3S_4$@NF before and after SOR stability test in 1 M $Na_2S$ + 1 M NaOH, HER in 1 M NaOH, and HER in 1 M NaOH seawater.

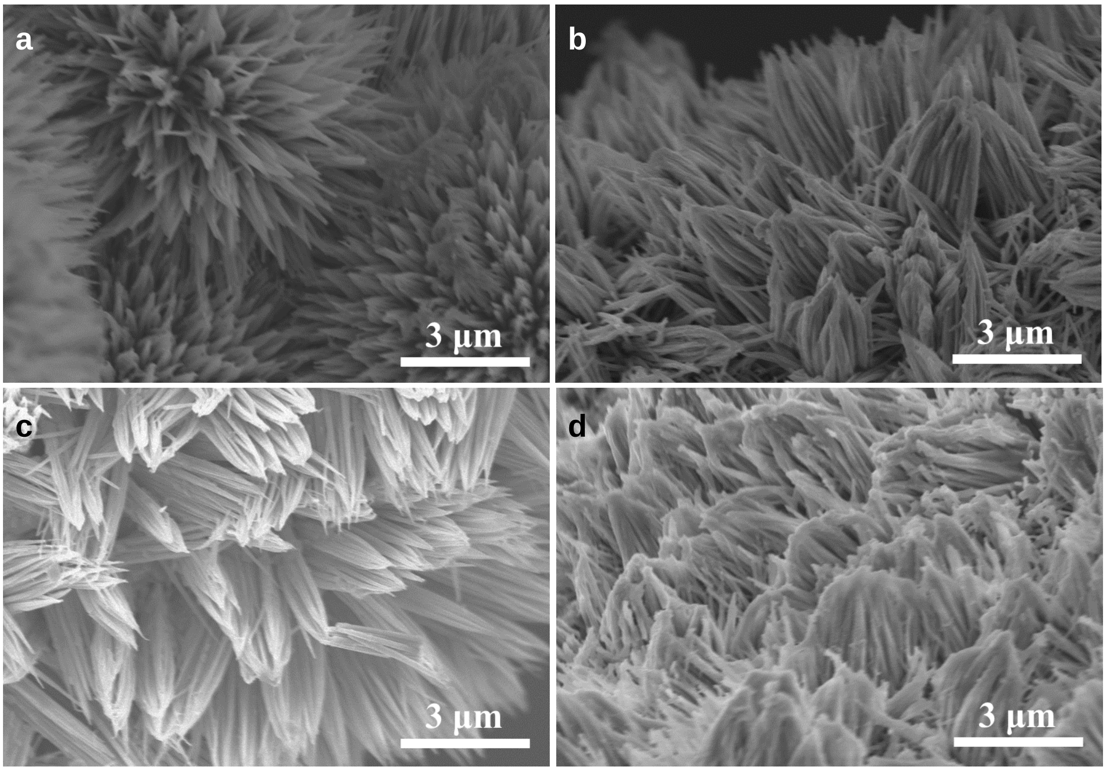


Supplementary Figure 20 | SEM images of *n*-$Co_3S_4$@NF (a) before and (b) after SOR stability test in 1 M $Na_2S$ + 1 M NaOH. SEM images of *n*-$Co_3S_4$@NF after HER stability test in (c) 1 M NaOH and (d) 1 M NaOH seawater.

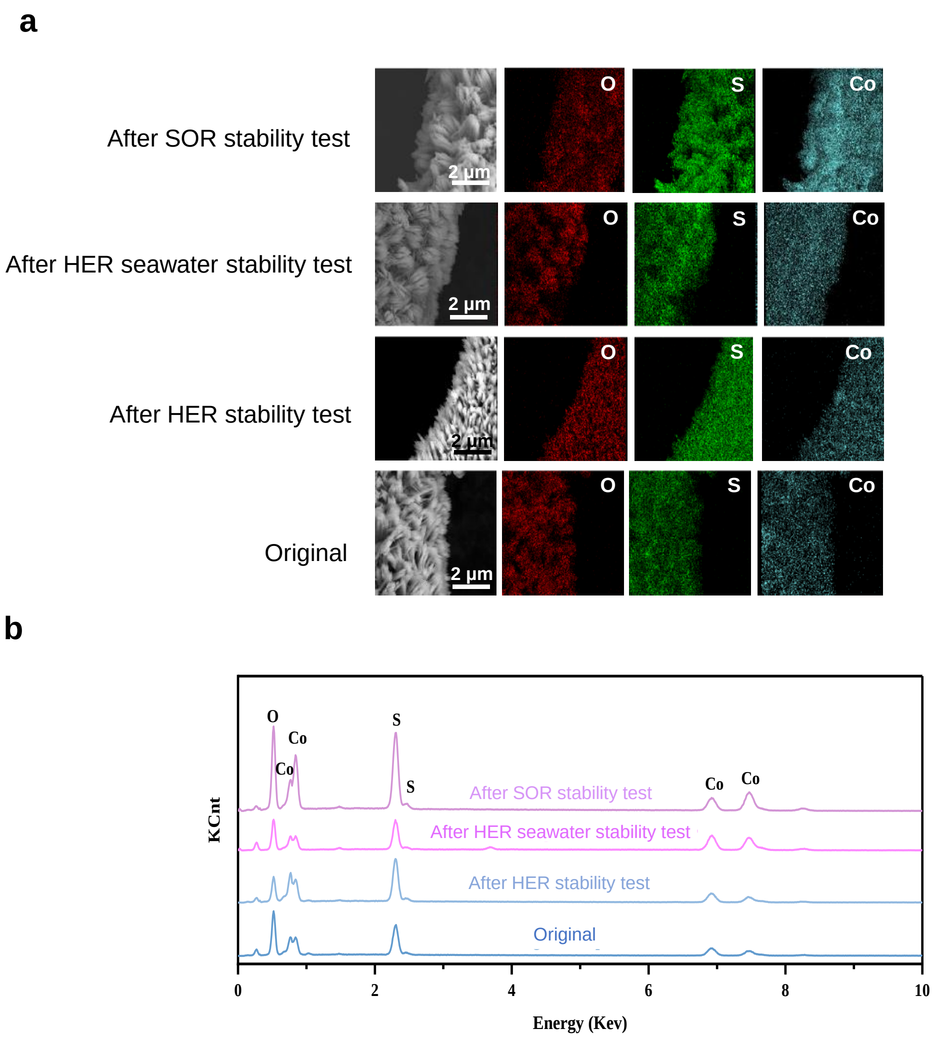


Supplementary Figure 21 | Before and after SOR stability test, HER seawater stability test, and HER stability test of (a) EDS elemental mapping images and (b) Typical EDS spectra.

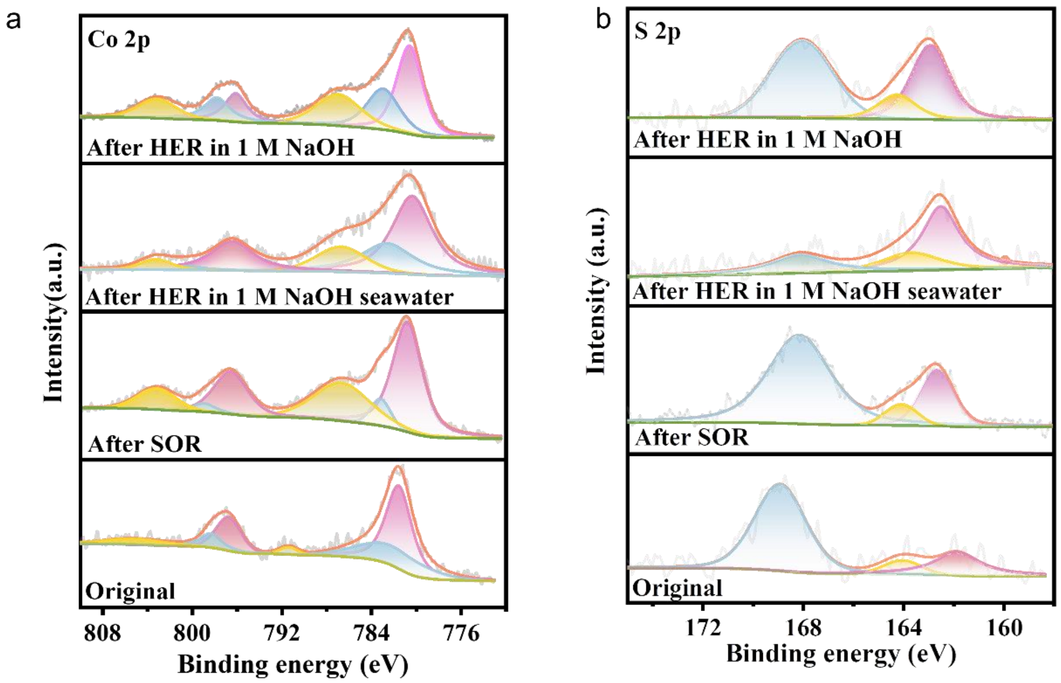


Supplementary Figure 22 | (a) High-resolution Co 2*p* XPS spectrum of *n*-$Co_3S_4$@NF before and after SOR stability test in 1 M $Na_2S$ + 1 M NaOH, HER stability test in 1 M NaOH and HER stability test in 1 M NaOH seawater. (b) High-resolution S 2*p* XPS spectrum of *n*-$Co_3S_4$@NF before and after SOR stability test in 1 M $Na_2S$ + 1 M NaOH, HER stability test in 1 M NaOH and HER stability test in 1 M NaOH seawater.

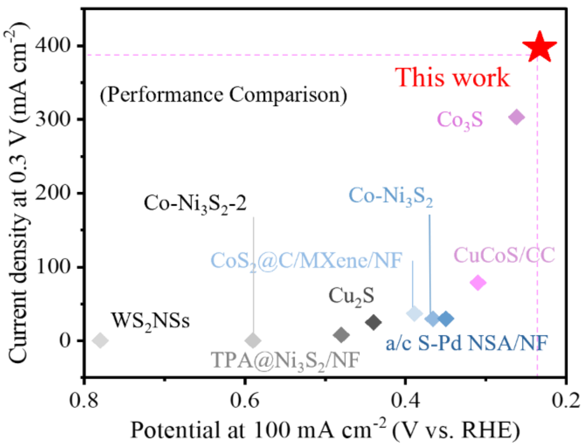


Supplementary Figure 23 | A comparison of $n$-$Co_3S_4$@NF with reported electrodes in electrocatalytic SOR performance.

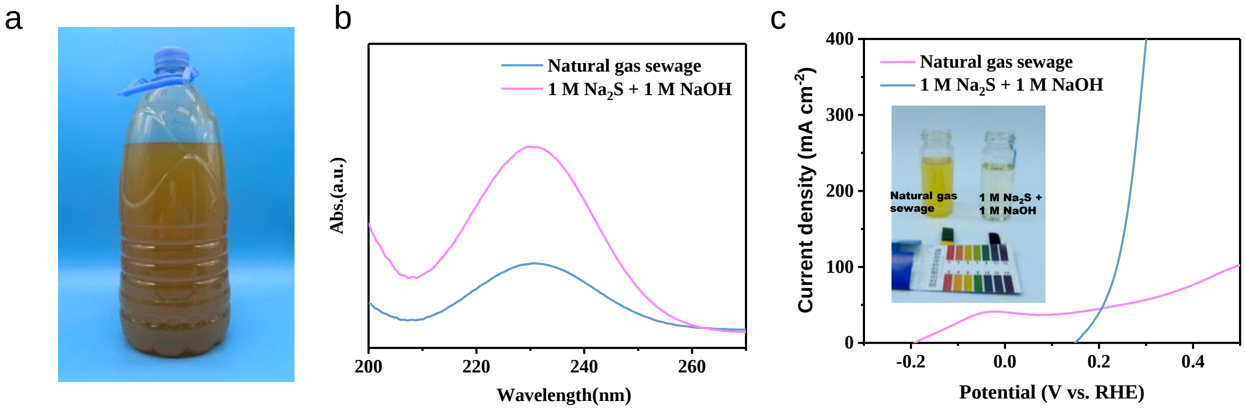


Supplementary Figure 24 | A demo for the *n*-$Co_3S_4$@NF used as SOR electrocatalyst for the purification of realistic sulfion-containing sewage form a natural gas field. (a) The optical photograph of the sulfion-containing sewage collected from Dazhou, Sichuan Province, PRC. (b) UV-vis spectra of different solutions. (c) The LSV curves for the SOR over *n*-$Co_3S_4$@NF in different reaction solutions, including the collected realistic sulfion-containing sewage and 1 M $Na_2S$ + 1 M NaOH as a blank control.

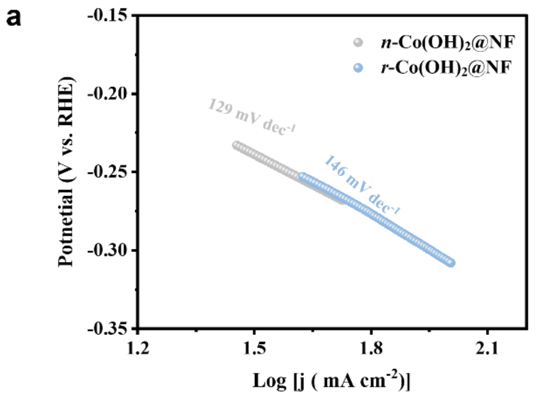


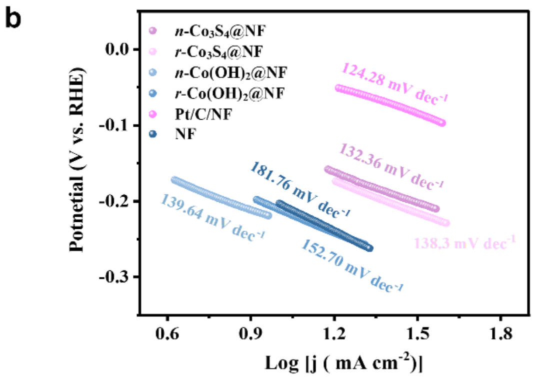


Supplementary Figure 25 | Tafel plots of catalysts for HER in (a) 1 M NaOH and (b) 1 M NaOH seawater.

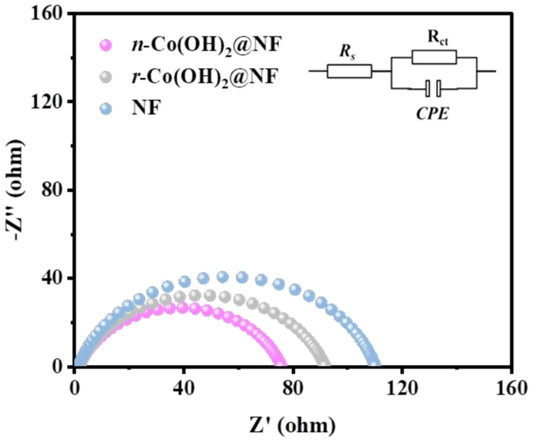


Supplementary Figure 26 | EIS plots of *n*-$Co(OH)_2$@NF, *r*-$Co(OH)_2$@NF, and NF for HER in 1 M NaOH.

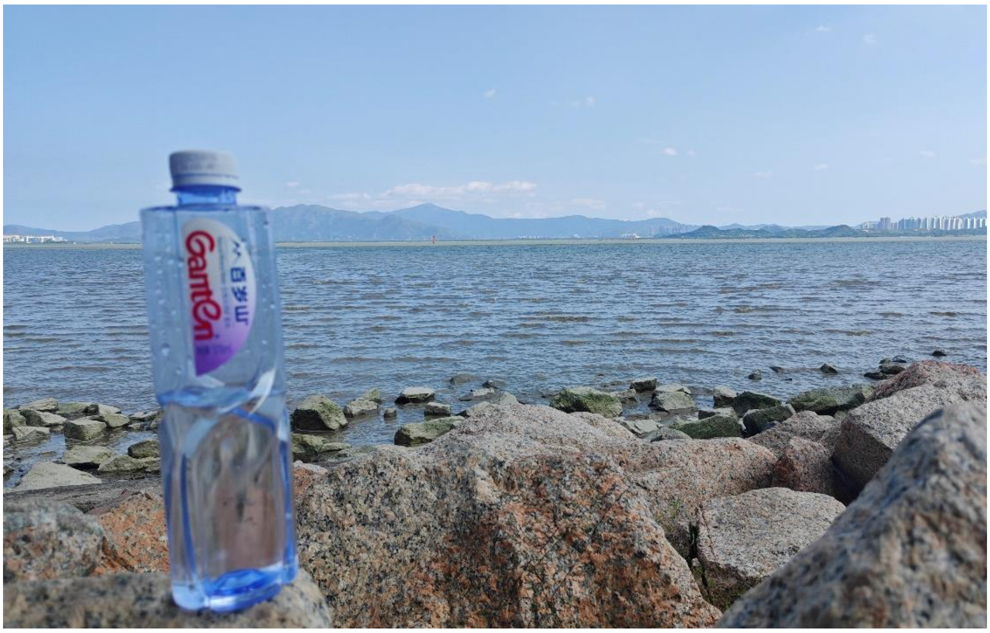

Supplementary Figure 27 | The optical photograph of seawater collected from Shenzhen Bay Park in Shenzhen, PRC.

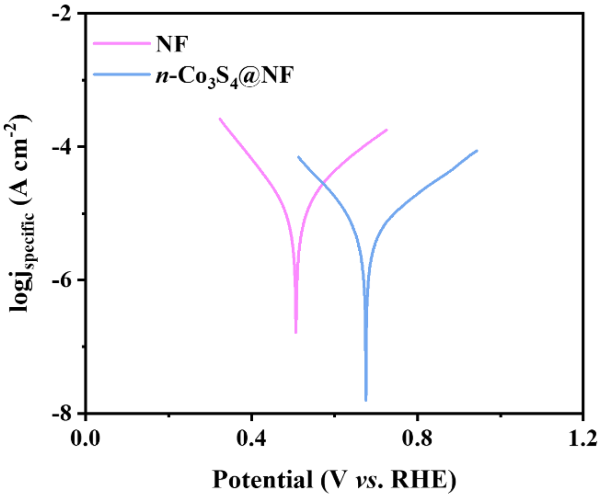


Supplementary Figure 28 | Tafel plots of *n*-$Co_3S_4$@NF and Ni foam in a 1 M NaOH seawater electrolyte.

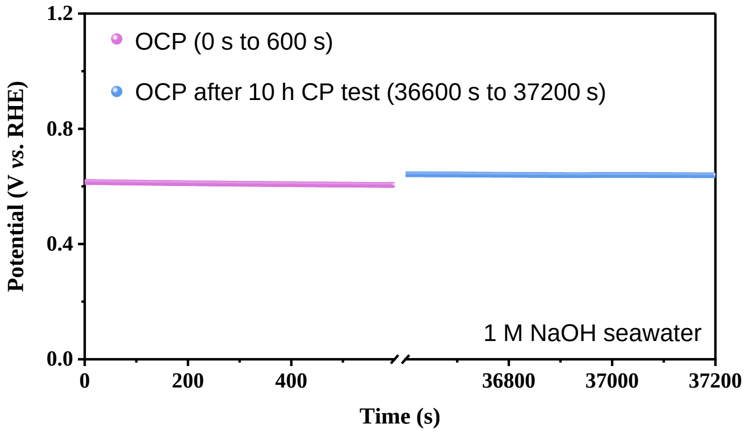


Supplementary Figure 29 | Open circuit potential (OCP) of *n*-$Co_3S_4$@NF before and after 10 h stability test in 1 M NaOH seawater at a current density of 100 mA $cm^{-2}$.

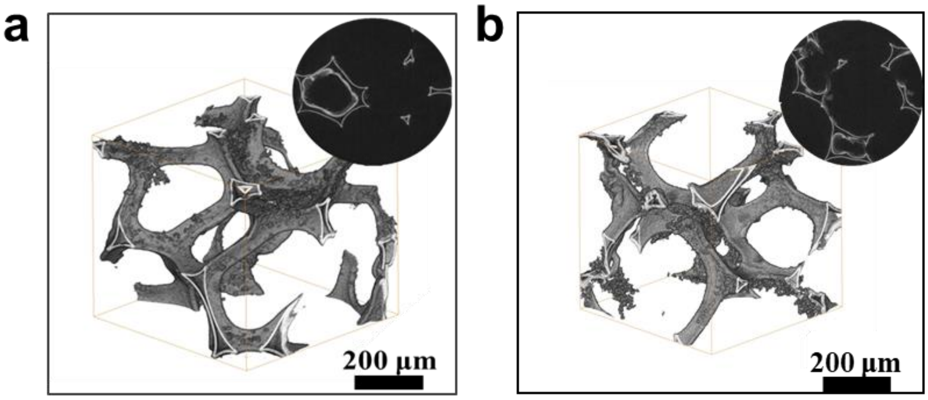


Supplementary Figure 30 | Three-dimensional X-ray tomography images of *n*-$Co_3S_4$@NF electrodes (a) before and (b) after 210 h HER stability test at the current density of 100 mA $cm^{-2}$ in 1 M NaOH seawater.

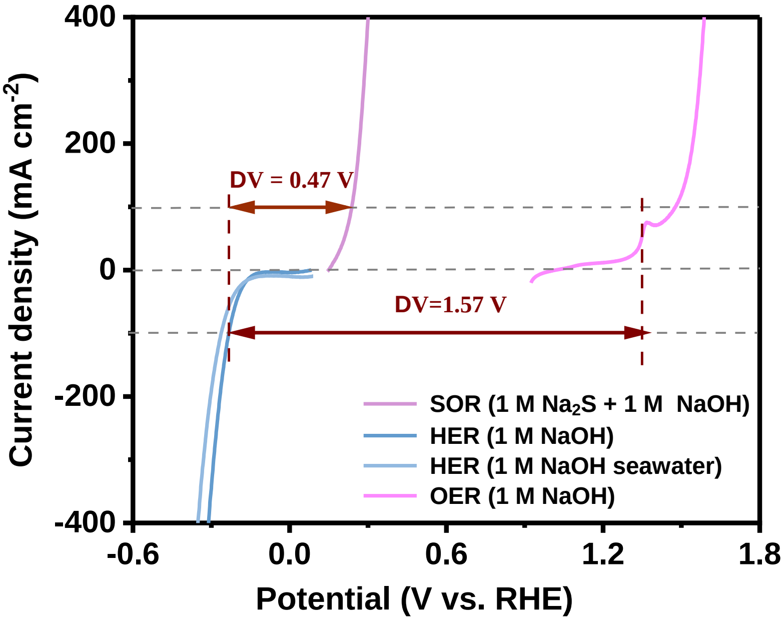


Supplementary Figure 31 | The voltage differences (ΔV) between HER and SOR or OER on *n*-$Co_3S_4$@NF in different electrolytes.

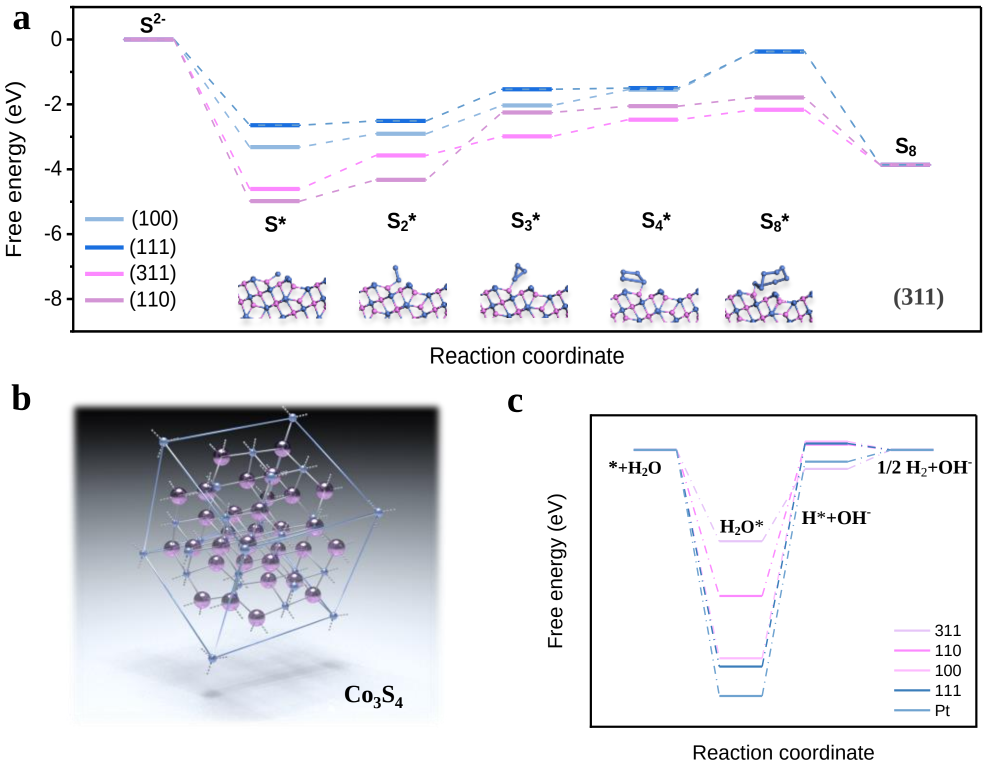


Supplementary Figure 32 | (a) Free energy profiles of stepwise SOR on different facets of $Co_3S_4$, insets represent the corresponding structural evolution of reaction intermediates adsorbed on the (311) facet of $Co_3S_4$. (b) Schematic illustration of $Co_3S_4$. (c) Free energy diagram of the HER catalyzed by $Co_3S_4$.

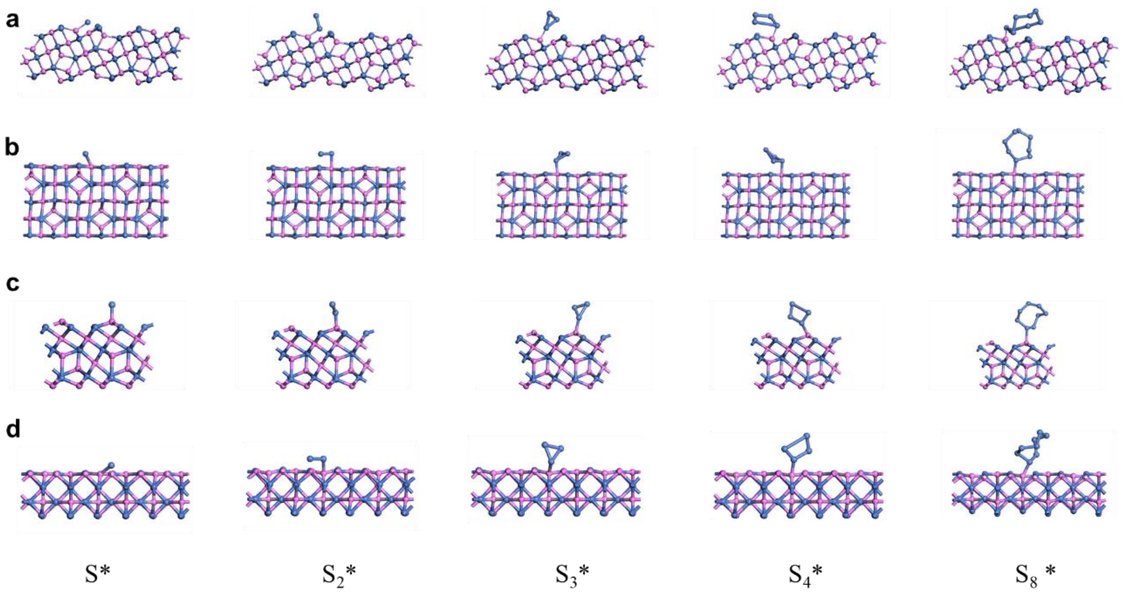


Supplementary Figure 33 | Structural models of reaction intermediates adsorbed on the (a) (311) facet, (b) (100) facet, (c) (111) facet, and (d) (110) facet of $Co_3S_4$.

Supplementary Figs. 32 and 33 via DFT calculations illustrate the stepwise oxidation mechanism of SOR on $Co_3S_4$ in a diagrammatic manner. Supplementary Fig. 32a shows that the (311) facet exhibits the lowest ΔG (1.03 eV), indicating that the (311) facet of $Co_3S_4$ has an excellent SOR activity propulsion capability. [1] Supplementary Fig. 32c revealed that the $Co_3S_4$ (311) has a high free energy value (ΔGH*) of -6.19 eV, indicating strong adsorption, moreover, the ΔGH* of the (110) facet of $Co_3S_4$ is 0.15 eV, indicating that the (110) facet can be implemented within the optimization of hydrogen bonding more efficiently, which further improves the kinetics of hydrogen desorption on the catalyst surface. Therefore, our catalyst represents a highly promising candidate in the field of sewage sulfion removal and energy-saving seawater electrolysis for hydrogen production.

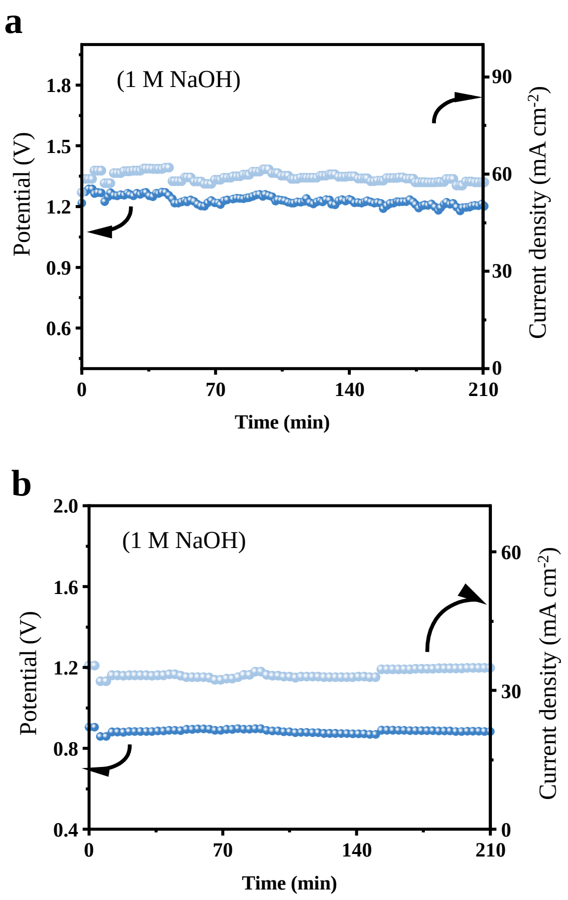


Supplementary Figure 34 | (a) The current density or voltage versus time curves of this TEG-driven HSE in 1 M NaOH, (b) The current density or voltage versus time curves of this solar cell driven HSE in 1 M NaOH.

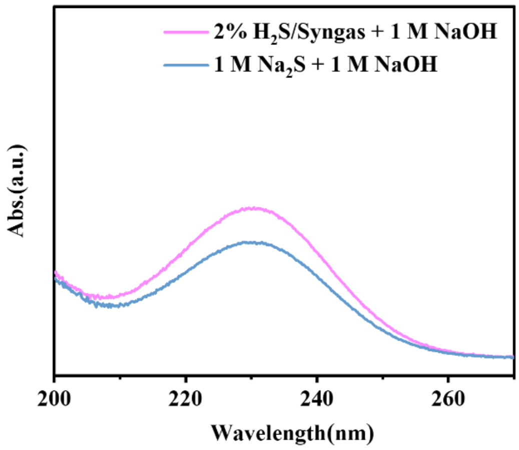


Supplementary Figure 35 | UV-vis spectra for 2% $H_2S$/syngas + 1 M NaOH and 1 M $Na_2S$ + 1 M NaOH.

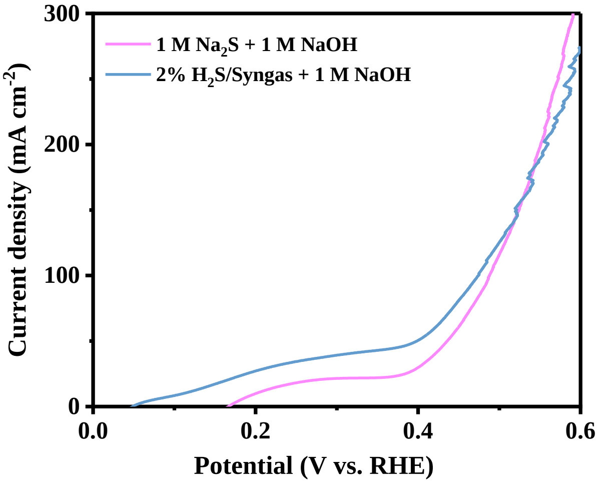


Supplementary Figure 36 | SOR polarization curves of *n*-$Co_3S_4$@NF in different electrolytes.

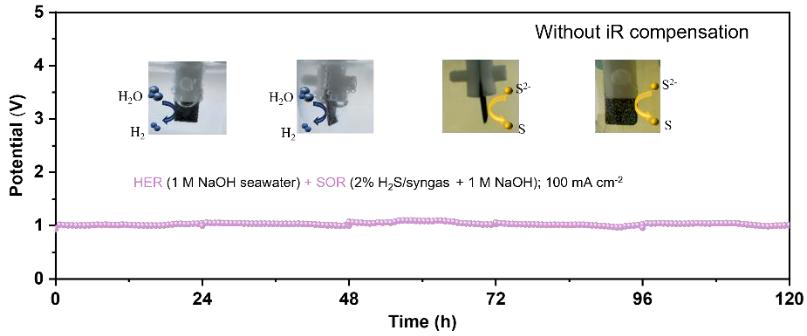


Supplementary Figure 37 | Durability measurement of $n$-$Co_3S_4$@NF for removing $H_2S$ in industrial syngas via 1 M NaOH solution with 2% $H_2S$/syngas. The measurement was carried out at a galvanostatic current of 100 mA $cm^{-2}$, and the corresponding potential of $n$-$Co_3S_4$@NF was maintained at around 0.8 V for 120 h. The fresh electrolytes were changed every 24 h.

Supplementary Table 1 | Comparison of the OER activity of the *n*-$Co_3S_4$@NF electrode to that of other recently reported in 1.0 M NaOH.

| **Catalytic electrode** | **OER overpotential@ $j_{100}$ (mV)** | **Reference** |
|---|---|---|
| P-$CoNi_2S_4$ | 1557 | [2] |
| CoNi@CNCoNiMoO | 1533 | [3] |
| N–Mo–Ni/NF | 1590 | [4] |
| NF@NiFe-LDH-1.5-4 | 1420 | [5] |
| (Mn,Ni)O(OH) TSNF | 1710 | [6] |
| NiFe-LDH/NFS-3h | 1469 | [7] |
| NF-S0.15 | 1478 | [8] |

Supplementary Table 2 | Comparison of the activities of different SOR electrocatalysts.

| **Catalysts** | **Current density (mA cm$^{-2}$) at 300 mV (*vs.* RHE)** | **Potential (mV *vs.* RHE) at 100 mA cm$^{-2}$** | **Support** | **Stability (h)** | **Electrolyte** | **Reference** |
|---|---|---|---|---|---|---|
| Co-$Ni_3S_2$ | 29.72 | 366 | Nickel foam | 64 h | 1.0 M NaOH+1.0 M $Na_2S$ | 9 |
| TPA@$Ni_3S_2$/NF | 7.88 | 483 | Nickel foam | 36 h | 1.0 M NaOH+1.0 M $Na_2S$ | 10 |
| CuCoS/CC | 78.81 | 312 | Carbon cloth | 20 h | 1.0 M NaOH+4.0 M $Na_2S$ | 11 |
| a/c S-Pd NSA/NF | 29.96 | 352 | Nickel foam | 20 h | 1.0 M KOH+3.0 M $Na_2S$ | 12 |
| $CoS_2$@C/MXene/NF | 36.81 | 389 | Nickel foam | 240 h | 1.0 M NaOH+1.0 M $Na_2S$ | 13 |
| $WS_2$ NSs | 0.38 | 783 | Carbon paper | 192 h | 1.0 M NaOH+1.0 M $Na_2S$ | 14 |
| $Co_3S_4$ | 303.12 | 262 | Nickel foam | 72 h | 1.0 M NaOH+1.0 M $Na_2S$ | 15 |
| $Cu_2S$ | 25.13 | 446 | Nickel mesh | 24 h | 1.0 M NaOH+1.0 M $Na_2S$ | 1 |
| Co-$Ni_3S_2$-2 | 0.20 | 592 | Nickel foam | 48 h | 1.0 M NaOH+1.0 M $Na_2S$ | 16 |
| *n*-$Co_3S_4$@NF | 397.19 | 233 | Nickel foam | 504 h | 1.0 M KOH+1.0 M $Na_2S$ | This work |

All catalysts were tested in room temperature.

Supplementary Table 3 | Calculation details of energy equivalent input and $CO_2$ equivalent emission for HSE and alkaline water electrolysis

| | Alkaline water electrolysis | | HSE | |
|---|---|---|---|---|
| Content | $CO_2$ equivalent emission ($t_{CO_2}$ $t_{H_2}^{-1}$) | Energy equivalent input (GJ $t_{H_2}^{-1}$) | $CO_2$ equivalent emission ($t_{CO_2}$ $t_{H_2}^{-1}$) | Energy equivalent input (GJ $t_{H_2}^{-1}$) |
| Water extraction & deionization | 0.045 | 0.000117 | 0 | 0 |
| Heating $H_2O$ to 70°C | 0.01 | 1.696 | 0 | 0 |
| Electrolysis stack | 1.04 | 187.2 | 0.272 | 48.9 |
| Heat demand of reaction | 0.151 | 27.25 | 0 | 0 |
| Overall | 1.246 | 216.146 | 0.272 | 48.9 |

Supplementary Table 4 | Comparison of the *n*-$Co_3S_4$@NF assembled hybrid seawater electrolyzer system with previously reported overall water splitting systems in cell performance.

| **Cell system** | **Electrolyte** | **Cell voltage at 100 mA $cm^{-2}$ (mV)** | **Power consumption (kWh $m^{-3}$ $H_2$)** | **Reactions** | **Reference** |
|---|---|---|---|---|---|
| *n*-$Co_3S_4$@NF(+, -) | 1.0 M NaOH +1.0 M $Na_2S$ (+) ‖1.0 M NaOH seawater (-) | 506 | 1.21 | HER/SOR | This work |
| *n*-$Co_3S_4$@NF (+, -) | 1.0 M NaOH seawater | 1574 | 3.77 | HER/OER | This work |
| $CoS_2$@C/MXene/NF (+) ‖ CoO@C/MXene/NF (-) | 1.0 M NaOH + 1.0 M $Na_2S$ (+) ‖ 1.0 M NaOH + seawater (-) | 590 | 1.41 | HER/SOR | [13] |
| $WS_2$ NSs/CP (+, –) | 1.0 M NaOH + 2.0 M $Na_2S$ (+) ‖ $H_2O$ ‖ 2.5 M $H_2SO_4$ (-) | 1321 | 3.16 | HER/SOR | [14] |
| $Ni_2P$-$Fe_2P$ (+, -) | 1.0 M KOH seawater | 1800 | 4.31 | HER/OER | [17] |
| Mo-$Ni_3S_2$ (+, -) | 1.0 M KOH | 1963 | 4.70 | HER/OER | [18] |
| $MoO_2$-FeP@C (+, –) | 1.0 M KOH + 1.0 M HMF (+) ‖ 1.0 M KOH (–) | 1700 | 4.07 | HER/HMFOR | [19] |
| Pt-$Co_3O_4$/CP (+, –) | 2.0 M MeOH + 1.0 M NaOH + 3.5% NaCl (+, –) | 1080 | 2.58 | HER/MOR | [20] |
| RP-CPM (+, -) | 1.0 M KOH + 0.3 M $N_2H_4$ (+) ‖ 1.0 M KOH (-) | 557 | 1.33 | HER/HzOR | [21] |
| Fe-$CoS_2$ (+, –) | 1.0 M KOH + 0.1.0 M $N_2H_4$ (+, –) | 610 | 1.46 | HER/HzOR | [22] |
| CoNi@CN-CoNiMoO (+, –) | 1.0 M KOH+ 0.5 M urea (+, –) | 1395 | 3.34 | HER/UOR | [3] |
| $NiFeO_x$-NF (+, –) | 1.0 M KOH + 0.5 M glucose (+) ‖ 1.0 M KOH (–) | 1390 | 3.33 | HER/GOR | [23] |
| $Fe_{0.1}$-$CoSe_2$/CC (+, –) | 1.0 M KOH + 0.5 M glucose (+) ‖ 0.5 M $H_2SO_4$ (–) | 1680 | 4.02 | HER/GOR | [24] |
| NC@$CuCo_2N_x$/CF (+, –) | 1.0 M KOH + 0.5 M benzyl alcohol (+) ‖ 1.0 M KOH (–) | 1825 | 4.37 | HER/BOR | [25] |

Supplementary Table 5 | The operational cost breakdown of the HSE and ASE in the ideal scenario.

| Parameters | HSE | ASE |
|---|---|---|
| Production rate (tones $H_2$/day) | 10 | 10 |
| Electrolyzer cost ($/kW) | 450 | 450 |
| Faradaic efficiency (%) | 99.1 | 99.1 |
| Capacity factor | 0.8 | 0.8 |
| Price ($ $m^{-2}$) | 10,000 | 10,000 |
| Cell voltage (mV) | 506 | 1574 |
| Current density (mA $cm^{-2}$) | 100 | 100 |
| System lifetime (year) | 20 | 20 |
| Discount Rate (%) | 7 | 7 |
| Electrolyte lifetime (year) | 1 | 1 |

* The input parameters are obtained from Ref. [26,27,28,29,30]

**Supplementary Note.** To determine the economic potential of hydrogen production from HSE, we carried out a simplified techno-economic analysis (TEA) based on a modified model.[26,27,29,30] Table S4 summarizes all inputs used in the sample calculation with lab data conditions.

Below is the list of assumptions made for the calculations.

1. The production capacity of the plant is 10 tones of hydrogen per day.
2. The electrolyzer cost of AWE is 300 $ $kW^{-1}$ obtained from the 2020 DOE target. Considering the configuration of the HSE, the electrolyzer cost of 450 $ $kW^{-1}$ is selected.
3. The total catalyst and membrane cost is 5% of the electrolyzer cost.
4. The capacity factor is expected to be operational on any given day, is assumed to be 0.8, which means the plant will be operational 292 days a year.
5. The price of electricity is assumed to be $ 0.02 $kWh^{-1}$, taken from recent onshore wind power auctions.[28]
6. Capital cost includes the electrolyzer cost and catalyst and membrane cost.
7. Balance of Plant are assumed to be 50% of the Capital cost.
8. Other operation and maintenance costs are assumed to be 10% of the Capital cost.
9. The faradaic efficiency for hydrogen generation is assumed to be 99.1%.
10. Due to the use of wastewater and seawater, the water consumption in the electrolyte is 0.

The calculation process

1. Electrolyzer cost

$$\text{Total electrolyzer cost} = \text{Power consumed} \times \frac{\text{base current density}}{\text{input current density}}$$

$$\text{Power consumed} = \text{Total current needed} \times \text{cell voltage}$$

$$\text{Total current needed} = \frac{\text{Plant capacity} \times \text{electrons transferred} \times \text{Faradaic constant}}{\text{Faradaic efficiency}}$$

$$\text{Capital recovery factor (CRF)} = \frac{\text{Discount rate} \times (1 + \text{Discount rate})^{\text{Lifetime}}}{(1 + \text{Discount rate})^{\text{Lifetime}} - 1}$$

$$\text{Electrolyzer cost} = \frac{\text{Capital recovery factor} \times \text{Total electrolyzer cost}}{\text{Capacity factor} \times 365 \times \text{Plant capacity}}$$

2. Catalyst and membrane cost

$$\text{Catalyst and membrane cost} = \frac{\text{Capital recovery factor}_{C\&M} \times \text{Total electrolyzer cost} \times 5\%}{\text{Capacity factor} \times 365 \times \text{Plant capacity}}$$

3. Maintenance and Other operation costs

Maintenance and Other operation costs = 10% × (Capital cost)

4. Balance of plant cost

Balance of plant cost = 50% × (Capital cost)

5. Electricity cost

$$\text{Electricity cost} = \frac{\mathit{Power\ consumed} \times 24 \times \text{Electricity cost}}{\text{Plant capacity}}$$

## References


1. Pei Y, Cheng J, Zhong H, Pi Z, Zhao Y, Jin F. Sulfide-oxidation-assisted electrochemical water splitting for $H_2$ production on a bifunctional $Cu_2S$/nickel foam catalyst. *Green Chem.* **23**, 6975-6983 (2021).
2. Lu XF, Zhang SL, Sim WL, Gao S, Lou XWD. Phosphorized $CoNi_2S_4$ yolk-shell spheres for highly efficient hydrogen production via water and urea electrolysis. *Angew. Chem. Int. Ed. Engl.* **60**, 22885-22891 (2021).
3. Qian G, Chen J, Jiang W, Yu T, Tan K, Yin S. Strong electronic coupling of CoNi and N-doped-carbon for efficient urea-assisted $H_2$ production at a large current density. *Carbon Ener.*, e368 (2023).
4. Wan J*, et al.* Nitrogen-doped nickel-molybdenum oxide as a highly efficient electrocatalyst for benzyl alcohol oxidation. *Green Chem.* **24**, 4870-4876 (2022).
5. Li X, Liu C, Fang Z, Xu L, Lu C, Hou W. Ultrafast room-temperature synthesis of self-supported NiFe-layered double hydroxide as large-current-density oxygen evolution electrocatalyst. *Small* **18**, e2104354 (2022).
6. Wang Y, Li Y, Ding L, Tang Y, Ding J. An (Mn,Ni)O(OH) turing structural nanoscale film driving highly efficient urea oxidization reaction in alkali water, seawater and waste water. *Surf. Coat. Technol.* **408**, 126799 (2021).
7. Wan Z, Ma Z, Yuan H, Liu K, Wang X. Sulfur engineering on NiFe layered double hydroxide at ambient temperature for high current density oxygen evolution reaction. *ACS Appl. Energy Mater.* **5**, 4603-4612 (2022).
8. Liao H*, et al.* Unveiling role of sulfate ion in nickel-iron (oxy)hydroxide with enhanced oxygen-evolving performance. *Adv. Funct. Mater.* **31**, 2102772 (2021).
9. Gao T*, et al.* Surface reconstructing hierarchical structures as robust sulfion oxidation catalysts to produce hydrogen with ultralow energy consumption. *Inorg. Chem. Front.* **10**, 1447-1456 (2023).
10. Jin L*, et al.* Ligand-induced electronic structure and morphology regulation in $Ni_3S_2$ heterostructures for efficient bifunctional electrocatalysis. *Appl. Surf. Sci.* **605**, 154756 (2022).
11. Yu H*, et al.* Electrocatalytic sulfion recycling assisted energy-saving hydrogen production using CuCo-based nanosheet arrays. *J. Mater. Chem. A* **11**, 2218-2224 (2023).
12. Wang W*, et al.* Sulfur-induced low crystallization of ultrathin Pd nanosheet arrays for sulfur ion degradation-assisted energy-efficient $H_2$ production. *Small* **19**, e2207852 (2023).
13. Zhang L, Wang Z, Qiu J. Energy-saving hydrogen production by seawater electrolysis coupling sulfion degradation. *Adv. Mater.* **34**, e2109321 (2022).
14. Yi L*, et al.* Scalable synthesis of tungsten disulfide nanosheets for alkali-acid electrocatalytic sulfion recycling and $H_2$ Generation. *Angew. Chem. Int. Ed.* **60**, 21550-21557 (2021).
15. Xiao Z*, et al.* Bifunctional $Co_3S_4$ nanowires for robust sulfion oxidation and hydrogen generation with low power consumption. *Adv. Funct. Mater.* **33**, 2212183 (2022).
16. Li Y, Duan Y, Zhang K, Yu W. Efficient anodic chemical conversion to boost hydrogen evolution with low energy consumption over cobalt-doped nickel sulfide electrocatalyst. *Chem. Eng. J.* **433**, 134472 (2022).
17. Wu L*, et al.* Heterogeneous bimetallic phosphide $Ni_2P$-$Fe_2P$ as an efficient bifunctional catalyst for water/seawater splitting. *Adv. Funct. Mater.* **31**, 2006484 (2020).
18. Du C, Men Y, Hei X, Yu J, Cheng G, Luo W. Mo-doped $Ni_3S_2$ nanowires as high-performance electrocatalysts for overall water splitting. *ChemElectroChem* **5**, 2564-2570 (2018).
19. Yang G*, et al.* Interfacial engineering of $MoO_2$-FeP heterojunction for highly efficient hydrogen evolution coupled with biomass electrooxidation. *Adv. Mater.* **32**, e2000455 (2020).

20. Xiang K, *et al.* Bifunctional Pt-$Co_3O_4$ electrocatalysts for simultaneous generation of hydrogen and formate via energy-saving alkaline seawater/methanol Co-electrolysis. *J. Mater. Chem. A* **9**, 6316-6324 (2021).
21. Li Y, *et al.* Partially exposed $RuP_2$ surface in hybrid structure endows its bifunctionality for hydrazine oxidation and hydrogen evolution catalysis. *Sci. Adv.* **6**, eabb4197 (2020).
22. McCoy DE, Feo T, Harvey TA, Prum RO. Structural absorption by barbule microstructures of super black bird of paradise feathers. *Nat. Commun.* **9**, 1 (2018).
23. Liu WJ, *et al.* Efficient electrochemical production of glucaric acid and $H_2$ via glucose electrolysis. *Nat. Commun.* **11**, 265 (2020).
24. Zheng D, *et al.* Three-birds-with-one-stone electrolysis for energy-efficiency production of gluconate and hydrogen. *Appl. Catal., B* **277**, 119178 (2020).
25. Zheng J, *et al.* Hierarchical porous NC@CuCo nitride nanosheet networks: highly efficient bifunctional electrocatalyst for overall water splitting and selective electrooxidation of benzyl alcohol. *Adv. Funct. Mater.* **27**, 1704169 (2017).
26. Sisler J, *et al.* Ethylene Electrosynthesis: A comparative techno-economic analysis of alkaline vs membrane electrode assembly vs $CO_2$-CO-$C_2H_4$ Tandems. *ACS Energy Lett.* **6**, 997-1002 (2021).
27. De Luna P, Hahn C, Higgins D, Jaffer SA, Jaramillo TF, Sargent EH. What would it take for renewably powered electrosynthesis to displace petrochemical processes? *Science* **364**, eaav3506 (2019).
28. Jouny M, Luc W, Jiao F. General techno-economic analysis of $CO_2$ electrolysis systems. *Ind. Eng. Chem. Res.* **57**, 2165-2177 (2018).
29. Zhou H, *et al.* Electrocatalytic upcycling of polyethylene terephthalate to commodity chemicals and $H_2$ fuel. *Nat. Commun.* **12**, 4679 (2021).
30. Leow WR, *et al.* Chloride-mediated selective electrosynthesis of ethylene and propylene oxides at high current density. *Science* **368**, 1228-1233 (2020).
