# Supplementary material for: Energy-saving hydrogen production by seawater electrolysis coupling tip-enhanced electric field promoted electrocatalytic sulfion oxidation
Source: Nat Commun. 2024 Jul 22;15:6173. doi: 10.1038/s41467-024-49931-5 (PMC11263359; doi:10.1038/s41467-024-49931-5)
Supplement: Supplementary file 3 — Description of Additional Supplementary Files [file 41467_2024_49931_MOESM3_ESM.pdf]

## Description of Additional Supplementary Files

**Supplementary Movie 1:** Solar-powered hybrid seawater electrolyzer (HSE) system for hydrogen production under sunlight.
